# The separation between mRNA‐ends is more variable than expected

**DOI:** 10.1002/2211-5463.13877

**Published:** 2024-09-03

**Authors:** Nancy Gerling, J. Alfredo Mendez, Eduardo Gomez, Jaime Ruiz‐Garcia

**Affiliations:** ^1^ Institute of Physics Biological Physics Laboratory San Luis Potosi Mexico; ^2^ Institute of Physics Laboratory of Molecular Biophysics San Luis Potosi Mexico; ^3^ Cold Atoms Laboratory, Institute of Physics Universidad Autónoma de San Luis Potosí San Luis Potosí Mexico

**Keywords:** contour length, mRNA, phenotypic stability, RNA external loop, RNA secondary structure

## Abstract

Effective circularization of mRNA molecules is a key step for the efficient initiation of translation. Research has shown that the intrinsic separation of the ends of mRNA molecules is rather small, suggesting that intramolecular arrangements could provide this effective circularization. Considering that the innate proximity of RNA ends might have important unknown biological implications, we aimed to determine whether the close proximity of the ends of mRNA molecules is a conserved feature across organisms and gain further insights into the functional effects of the proximity of RNA ends. To do so, we studied the secondary structure of 274 full native mRNA molecules from 17 different organisms to calculate the contour length (*C*_L_) of the external loop as an index of their end‐to‐end separation. Our computational predictions show bigger variations (from 0.59 to 31.8 nm) than previously reported and also than those observed in random sequences. Our results suggest that separations larger than 18.5 nm are not favored, whereas short separations could be related to phenotypical stability. Overall, our work implies the existence of a biological mechanism responsible for the increase in the observed variability, suggesting that the *C*_L_ features of the exterior loop could be relevant for the initiation of translation and that a short *C*_L_ could contribute to the stability of phenotypes.

## Graphical abstract

Both mRNA ends are in proximity despite mRNAs origin, length and secondary or tertiary structures. However, we show that the distance between native mRNA ends is not constant and varies across organisms. We found that native sequences show much larger variations than those observed in random sequences, implying the existence of a biological mechanism responsible for the increased variability.

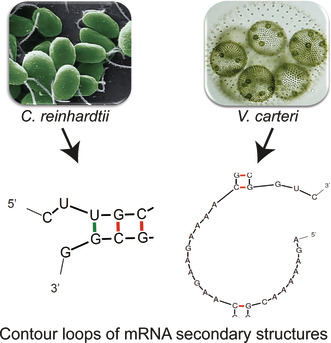

**Abbreviations**
*C*_L_
: contour length
FE: free energy
MFE: minimum free energy
nt: nucleotide
PABP: poly(A) binding protein
PAS: polyadenylation signal
UTR: untranslated region

Ribonucleic acid (RNA) molecules participate in several key cellular functions, for instance, messenger RNA (mRNA) molecules are templates for the synthesis of proteins; or perform by themselves functions such as catalysis (Ribozymes), RNA processing by splicing (snRNA), and the control of gene expression (miRNA and ncRNA), among others. To perform their functions and be recognized by proteins, RNA molecules fold into complex secondary and tertiary structures. This is achieved by the formation of base‐paired (double‐stranded) regions as well as of unpaired (single‐stranded) loops following the thermodynamics of base pairing in a hierarchical fashion [1], which leads to an increase in the conformational stability of RNA molecules [2, 3]. Importantly, the folding of any given RNA molecule is remodeled as it is transcribed, thus their different structures are dynamically changed as new domains of the RNA molecules appear in the scene [4]. In addition, the interaction with proteins also modify the folding transitions of the RNA structures [4]. Regardless of these, the 5′ and 3′ ends of RNA molecules are left loose in close proximity forming the so‐called exterior loop, providing an intrinsic circularization of RNA molecules.

The current predominant view is that the circularization of mRNAs is actually induced by their interaction with the translation initiation factor eIF4F (comprised of eIF4E, eIF4G and eIF4A) in the 5′ end, and with the poly(A) binding protein (PABP) in the 3′ end [5, 6, 7], resulting in the formation of a looped‐RNA stabilized by the bridging interaction of eIF4G with PABP [5, 6]. This induced‐effective circularization regulates in principle the rate‐limiting step of translation initiation [5]. However, several lines of evidence suggest instead that intramolecular structuring of RNA results in the close proximity of both ends of RNA molecules providing the effective circularization that could facilitate the initiation of translation. For instance, using Fluorescence Förster Resonance Energy Transfer (FRET) in free‐protein preparations of *in vitro* generated mRNA molecules fluorescently labeled at both ends, two groups showed that the distance between both ends of mRNA molecules is rather small, of 5–9 nm in some genes of the fungus *Trichoderma atroviride* [8] or < 7 nm in some housekeeping genes of human and *Saccharomyces cerevisiae* [9]. Therefore, this separation corresponds to the contour length (*C*_L_) of their exterior loop that falls between 9 and 16 nt, i. e., the unpaired bases of both 5′ and 3′. This constant proximity of both ends of RNA molecules was previously suggested by thermodynamic computational analysis [10]. Remarkably, this small and constant length of the separation between both ends seems to be independent of the nucleotide sequence, type of RNA molecule, origin or the size of the RNA, since monocistronic or bicistronic coding and non‐coding RNAs of different size from viral, fungal, or mammalian origin were experimentally determined [8, 9]. Moreover, disruption of the secondary structure near the exterior loop disturbs and delays translation initiation [11], and disruption of the eIF4G‐PABP interaction has no impact on the spatial organization of circularized RNA [12]. In addition, single‐molecule blotting experiments of cross‐linked endogenous polysomal fractions show little, if any, colocalization of all eIF4E, eIF4G and PABP with RNA molecules, suggesting an incompatibility between RNA circularization and active translation [13]. All of these raises the possibility that the features of *C*_L_ of the exterior loop could be relevant for the recruiting of the translation initiation machinery, independently of the MFE secondary structure adopted by the whole mRNA molecule.

However, the end‐to‐end distance of mRNA molecules have been studied only in a handful of species. We therefore decided to extend these observations to 274 full native mRNA molecules from 17 species reported to the GenBank to investigate whether this close proximity is present in other forms of life, and to explore whether there have some biological implications.

## Methods

### mRNA sequences

All analyses were performed using native full‐length transcriptional units reported to the GenBank Database (https://www.ncbi.nlm.nih.gov/genbank/). mRNA sequences were selected only if: (a) the reported sequence have the presence of both 5′‐ and 3′‐UTRs; (b) the 3′‐UTRs contained the polyadenylation signal and the start of the poly‐A tail; and (c) the length falls between 200 and 7000 nt including both UTRs and coding sequence. Out of 3000 mRNA sequences analyzed, only 274 mRNA sequences fulfilled all the requirements (Table S1), therefore, our work was limited by the availability of full native mRNA sequences. The selected organisms and the data for their native full mRNA sequences are shown in Table S1A. Identification of homologous genes (Table S1B) was performed by means of bioinformatic analysis (from the GenBank) at the protein level. The BLASTp alignment program (https://blast.ncbi.nlm.nih.gov/Blast.cgi) was then used to verify the presence of the homologous protein in their related species. However, given that full mRNA sequences for homologous genes across species are very scarce, to obtain their MFE secondary structures it was necessary in some cases to identify their complete mRNA sequences using their genome information by determining the transcription initiation site using the Inr element, as well as the polyadenylation site for each specie [14, 15, 16, 17]. For comparison, 50 random‐generated sequences of 1600 nt were generated with equal probability for the four nucleotides using a macro in the Excel program.

### Prediction of the distance between the ends of mRNA molecules

**Fig. 1:**
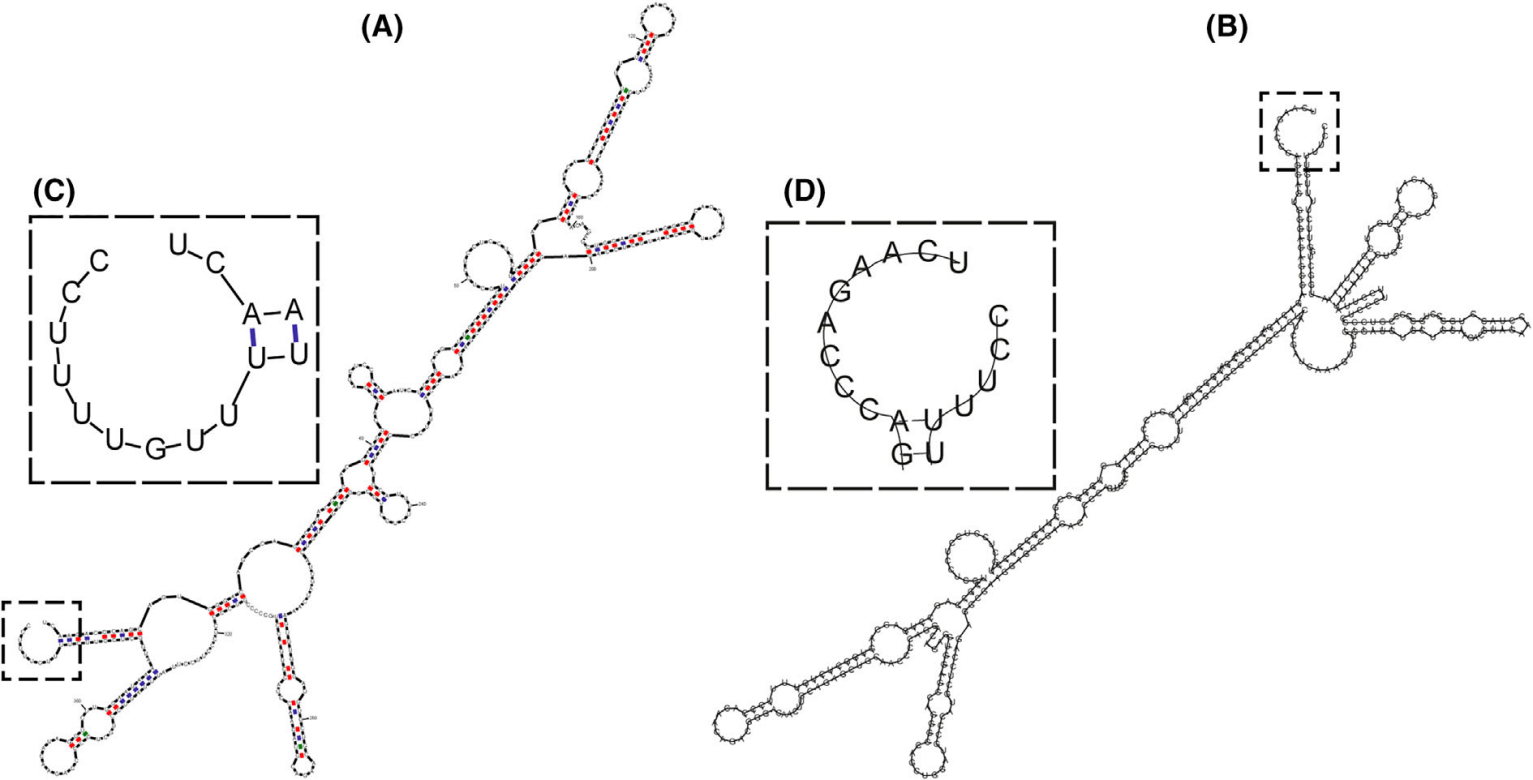
Minimum free energy secondary structure for 391 nt mRNA of *PT hepcidin (HAMP)*. Obtained by (A) mfold and (B) Vienna RNA programs. The exterior loop is quite similar despite differences on their secondary structure. (C, D) Zooms of the exterior loop of (A) and (B), respectively.

Prediction of the secondary structure of each mRNA was performed using both mfold [18] and Vienna RNA programs [19]. Then, to estimate the distance between the ends of each mRNA molecule, the contour length (*C*_L_) of the exterior loop from each secondary structure was obtained by counting the number of nucleotide links comprising the exterior loop multiplied by the typical distance (*d =* 0.59 nm) between nucleotides in RNA molecules [20] (Fig. S1). Although a degeneration in base pair prediction accuracy that increases with the length of sequence has been proposed [21, 22, 23], prediction of the secondary structure of a single sequence is reasonably accurate with the RNA folding programs we used here [24]. Moreover, different probabilistic models give similar results [25, 26, 27]. Indeed, regardless of the clear differences in the secondary structure, when compared, both programs gave quite similar results in the *C*_L_ values (Fig. 1, Figs S2–S4), which is the distance of interest in our work.

**Fig. 2:**
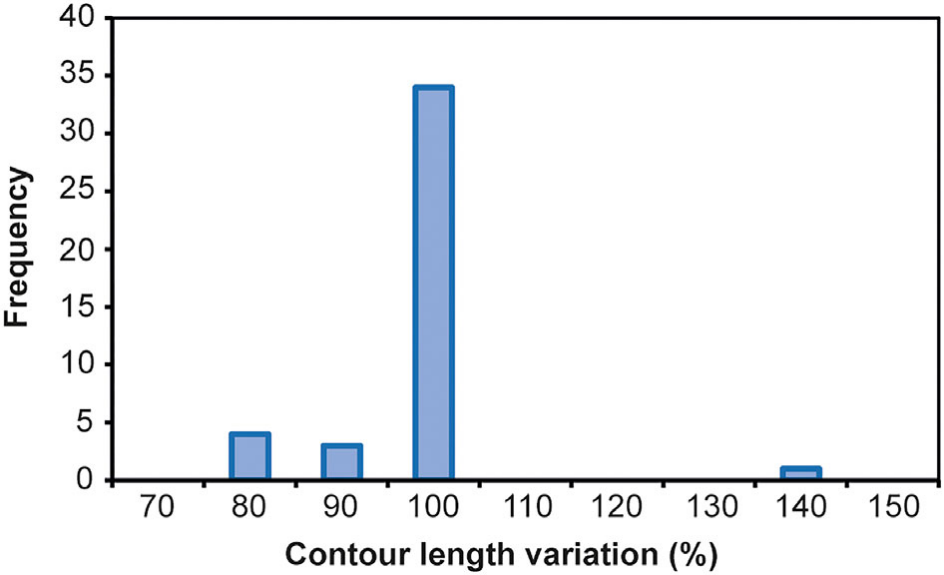
Contour length ratio between the minimum free energy and free energy structures. No significant differences appear between the MFE structure and the subsequent FE structures for the same mRNA, *P* = 0.1753 and *N* = 45.

Furthermore, it is well known that the mRNA molecule can fold into different secondary structures but when the *C*_L_ of 5 suboptimal structures (for the same RNA sequence) with the lowest free energy (FE) were compared against the *C*_L_ of the minimum free energy (MFE) secondary structures, no significant differences were found despite the differences in the secondary structure (*P =* 0.1753) (Fig. 2). This led us to suggest that the *C*_L_ remains stable and independent of the secondary structure that the molecule could adopt. For these reasons, and because in our study the mRNA secondary structure is totally irrelevant, only the *C*_L_ is the feature of interest, we decided to use the MFE secondary structure in our study (only to obtain the *C*_L_ values), which correspond to the thermodynamically most stable structure with the lowest possible energy (Δ*G*).

### Statistical analysis

All data presented are mean ± SEM (standard error of the mean) unless otherwise stated. Statistical differences were analyzed by the two‐tailed Welch's test for unequal variances. Differences were significant at *P* < 0.05. For the linear fits in our data, we used linear regression and two‐tailed significance tests at *P* < 0.05.

## Results

It has been demonstrated computational and experimentally that the ends of RNA molecules have an innate proximity, however using only a low number of RNA molecules [8, 9, 10]. We therefore decided to extend these observations by computationally determining the end‐to‐end distance of 274 full native mRNA molecules from 17 species reported to the GenBank. The species used for this study are: the halophilic archaeon *Halobacterium salinarum* (*HbS*); the single‐cell green alga *Chlamydomonas reinhardtii* (*CR*); the colonial green alga *Volvox carteri* (*VC*); the jawless fish *Lethenteron camtschaticum* (*LC*); the scorpion *Pandinus imperator* (*PI*); the cockroach *Blatella germanica* (*BG*); the scot pine *Pinus sylvestris* (*PS*); the maidenhair tree *Ginkgo biloba* (*GB*); the fowl *Gallus gallus* (*GG*); the opossum *Monodelphis domestica* (*MD*); the monocotyledon *Zea mays* (*ZM*); the dicotyledons rubber tree *Hevea brasiliensis* (*HB*); the flowering plant *Arabidopsis thaliana* (*AT*); the honey bee *Apis cerana* (*AC*); the fresh water jawed fish *Danio rerio* (*DR*) as well as the hominids *Pan troglodytes* (*PT*) and *Homo sapiens* (*HS*).

Both mfold and Vienna RNA programs were used to obtain the MFE secondary structures of full‐length native mRNAs randomly selected from these species. The end‐to‐end distance was determined by calculating the *C*_L_ of the exterior loop (Fig. S1). Remarkably, although clear differences in the whole secondary structures can be found with each program, both programs gave quite similar results for *C*_L_ values (Fig. 1, Figs S2–S4), thus, we chose to report here the values obtained with the Vienna RNA algorithm.

**Fig. 3:**
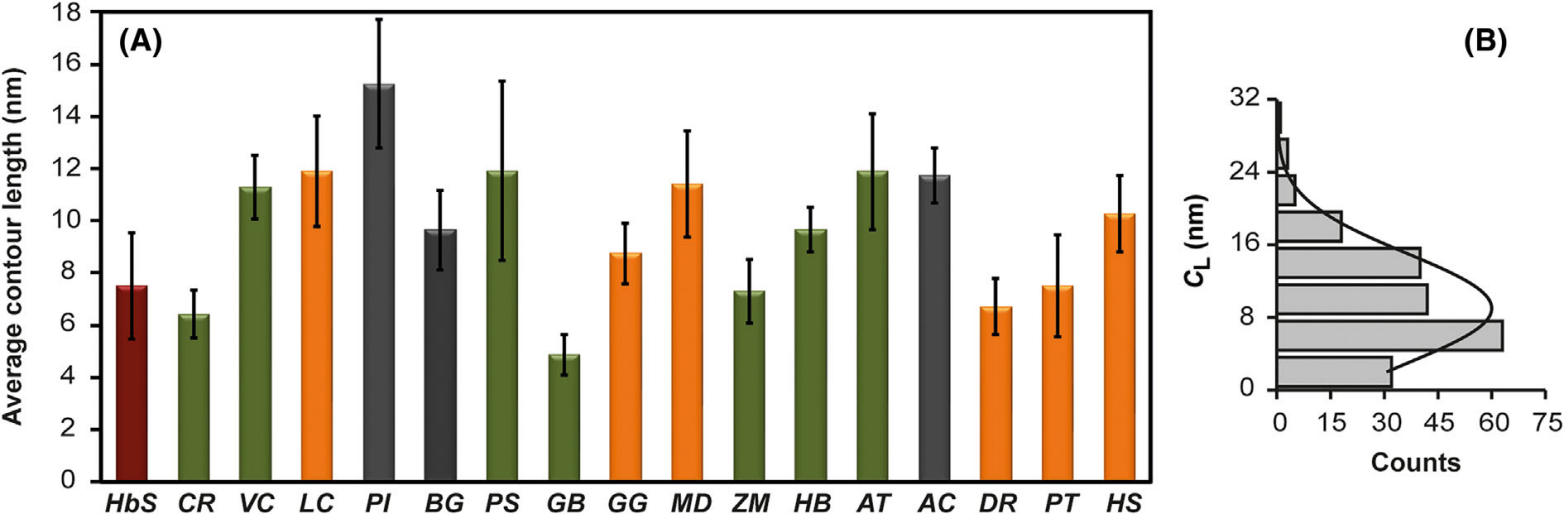
Contour length distributions from the predicted mRNA secondary structures. (A) Average contour length from the predicted mRNA secondary structures obtained by Vienna RNA predictions. *H. salinarum* (*HbS*), *C. reinhardtii* (*CR*), *V. carteri* (*VC*), *L. camtschaticum* (*LC*), *P. imperator* (*PI*), *B. germanica* (*BG*), *P. sylvestris* (*PS*), *G. biloba* (*GB*), *G. gallus* (*GG*), *M. domestica* (*MD*), *Z. mays* (*ZM*), *H. brasiliensis* (*HB*), *A. thaliana* (*AT*), *A. cerana* (*AC*), *D. rerio* (*DR*), *P. troglodytes* (*PT*) and *H. sapiens* (*HS*). The bars correspond to the average value of all mRNA used for each species. Bars with the same color represent organism of a same clade: halobacteria (red bars); Viridiplantae (green bars); invertebrates (gray bars) and vertebrates (orange bars). We include the standard error of the average of the mRNA molecules included per species with a total sample size of *N = 204*. (B) We show the histogram for all *C*_L_ values obtained with Gaussian fit (black line). We can observe that the mRNA molecules have a *C*_L_ smaller than 17.5 ± 2 nm (± SD) with 95% confidence level.

**Fig. 4:**
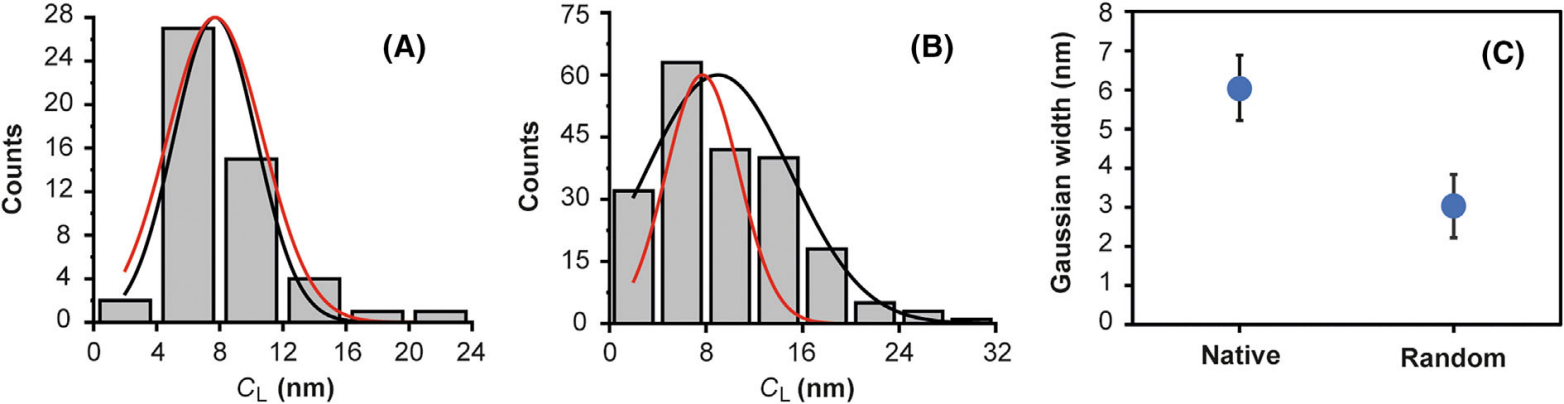
Difference between the contour length of random‐generated and native sequences. (A) Histogram obtained for the *C*_L_ values of random‐generated sequences with *N =* 50. We can observe that the sequences have a *C*_L_ smaller than 11.4 ± 1.4 nm with 95% confidence level. We show the Gaussian fit for not scaled (*w =* 2.6 ± 0.7 nm) and scaled values (*w =* 3.03 ± 0.81 nm) (black line and red line respectively) with the same amplitude for the comparison of both. (B) Histogram obtained for the *C*_L_ values of native sequences. We show the Gaussian fit for native (*w =* 6.03 ± 0.86 nm) and the scaled value for random‐generated sequences (*w =* 3.03 ± 0.81 nm) (black line and red line respectively), again with the same amplitude for the comparison. (C) We observe a higher variation between the native and the scaled value for random‐generated distribution widths with a 3.7 σ confidence. The data presented are ± SD.

The average contour length of the randomly selected full native mRNA sequences reported to the GenBank is shown in Fig. 3. On average, the *C*_L_ varies from 4.8 (*GB*) up to 15.2 nm (*PI*), which is wider than previously reported [8, 9, 10]. Moreover, as can be noted in Fig. 3, the separation between the ends of mRNA molecules do not remain constant. Indeed, when individually evaluated, the separation between mRNA ends varies from 0.59 up to 31.8 nm (Fig. 3B). The Gaussian fit is centered on *x =* 9.0 ± 0.8 nm and has a width of *w =* 6.03 ± 0.86 nm [± Standard Deviation (SD)]. The statistics give a *C*_L_ smaller than 17.5 ± 2 nm (± SD) (95% confidence level) thus larger *C*_L_ values are not favored. Therefore, as a control, we performed a similar analysis to emphasize the difference, using only randomly generated sequences (Fig. 4). The 50 sequences had 1600 nt with an equal probability for the four different nucleotides. The resulting histogram (Fig. 4A) shows that the *C*_L_ values varies from 4.1 up to 21.2 nm and the statistics give a *C*_L_ smaller than 11.4 ± 1.4 nm (± SD) with 95% confidence level. The Gaussian fit is centered at *x =* 7.7 ± 0.4 nm and has a width of *w =* 2.6 ± 0.7 nm (± SD). Comparing the width of the native (*w =* 6.03 ± 0.86 nm) (Fig. 4B) and random‐generated sequences (*w =* 2.6 ± 0.7 nm) we see that the native RNA sequences show considerably higher variations. This difference cannot be accounted for by the different value of the Gaussian center or by the variations in GC content (see Discussion about the GC dependence below). Scaling the width of the sequences randomly generated by these two factors would give *w =* 3.03 ± 0.81 nm (± SD) (Fig. 4A,B). Thus, the final probability to have these variations, due to a statistical fluctuation, is smaller than 0.01% [3.7 σ confidence (Fig. 4C)]. The above result indicates that there must be an underlying biological mechanism contributing to the selection of particular contour lengths, since the variations cannot be explained by thermodynamic reasons alone.

The biological implications of the end‐to‐end proximity have not yet been explored, and because our results point toward an underlying biological mechanism contributing to the selection of particular contour lengths, we decided to explore further some possible biological mechanisms.

The efficiency during translation initiation is particularly dependent on the effective circularization of mRNAs, so any increase in *C*_L_ would have serious implications on the rate of translation of any given mRNA. Intriguingly, the length of 3′‐UTRs shows an increase as the level of complexity of organisms increases, suggesting that 3′‐UTRs may have increased with evolution [28], and thus modifying the efficiency of translation. Moreover, phenotypical stability, which is the ability to maintain the same phenotype in response to environmental changes, depends on the efficiency of protein synthesis; conversely, low translation efficiencies could increase the opportunity for variability. As can be seen in Fig. 3, when the *C*_L_ values are plotted in order of the temporal range of their first ancestors' appearance [29, 30, 31, 32, 33, 34, 35, 36, 37, 38, 39, 40, 41, 42, 43, 44, 45, 46, 47], although no evolutionary trend could be found, the separation between the ends of mRNA molecules do not remain constantly small. For instance, insects (*BG* and *AC*) as well as mammals (*MD*, *PT* and *HS*), angiosperms (*ZM*, *HB* and *AT*) and fishes (*LC* and *DR*) have similar *C*_L_ among them, this is, no variability could be found when related species are evaluated. However, the multicellular green alga *VC* has a significantly longer *C*_L_ value than its closest evolutionary predecessor *CR* (*P =* 0.007). In contrast, the eudicotyledones *HB* and *AT*, which have a similar level phylogenetic divergence, present similar *C*_L_ values (9.7 ± 0.8 nm for *HB* versus 11.9 ± 2 nm for *AT*, *P =* 0.36), and somewhat similar with the hominids *PT* and *HS* (7.5 ± 2 versus 10.3 ± 1.5 nm, respectively, *P =* 0.27).

To have a better insight of any impact of the end‐to‐end separation of mRNA molecules on translation, the separation between the ends of mRNA molecules was analyzed in homologous (see Table S1B), housekeeping and highly expressed genes (see Table S1A).

**Fig. 5:**
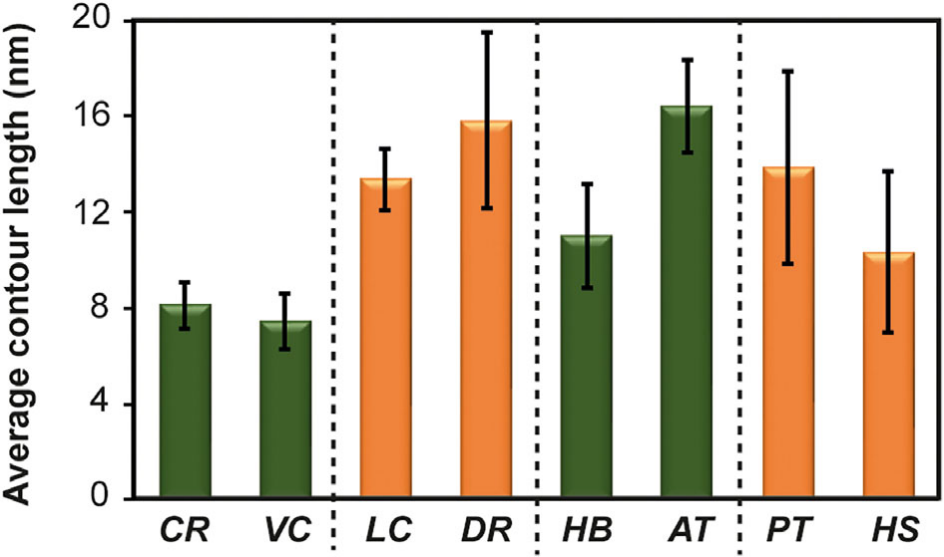
Contour length distributions from the predicted mRNA secondary structures in related species. Average contour length from the homologous genes in related species *C. reinhardti*i (*CR*), *V. carteri* (*VC*), *L. camtschaticum* (*LC*), *D. rerio* (*DR*), *H. brasiliensis* (*HB*), *A. thaliana* (*AT*), *P. troglodytes* (*PT*) and *H. sapiens* (*HS*). Black dashed lines divide the related species. Bars with the same color represent organism of a same clade: Viridiplantae (green bars) and vertebrates (orange bars). We include the standard error of the average of the mRNA molecules included per species with a total sample size of *N = *70. Statistical differences between the *C*_L_ values of related species were analyzed by the two tailed Welch's test for unequal variances.

As would be expected, when compared, homologous genes from related species (green algae *CR* and *VC*; fishes *LC* and *DR*; eudicots *HB* and *AT* and the hominids *PT* and *HS*), showed no significant differences (Fig. 5). However, when compared against heterologous genes, the *C*_L_ values of homologous, housekeeping and highly expressed genes, have lower *C*_L_ values, suggesting that constant level of expression at the protein level of these three types of genes could be related to a small end‐to‐end distance of their respective mRNA.

**Fig. 6:**
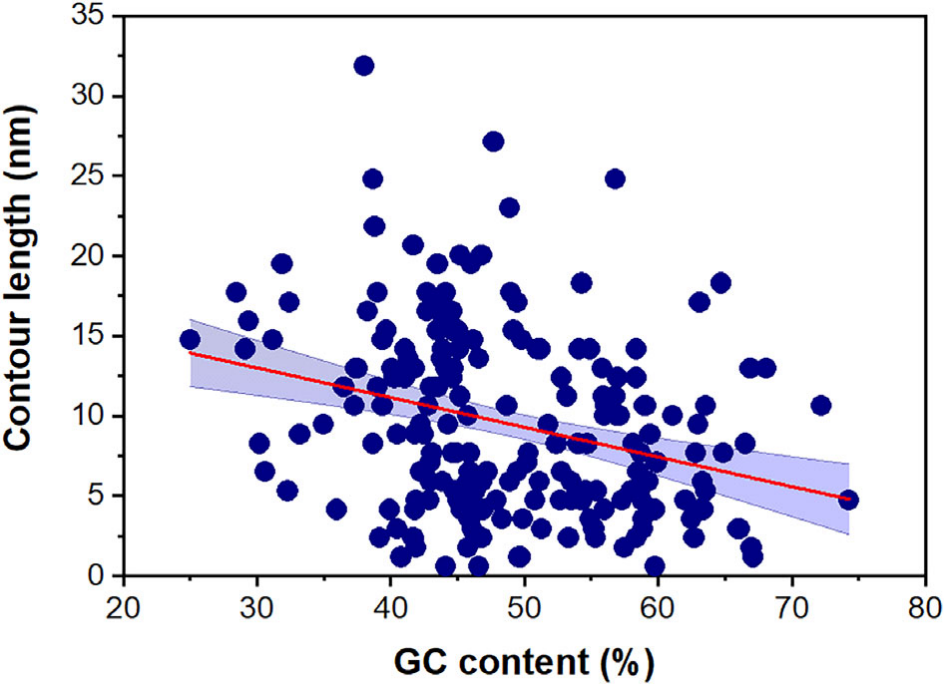
Contour length vs GC content from the predicted mRNA secondary structures. Blue circles correspond to values obtained using Vienna RNA, *N* = 204. The plot includes the linear fit (red line) (*y = a + bx*) with *a =* 18.6 ± 2 nm and *b =* −0.18 ± 0.04 nm/% GC. The Pearson correlation coefficient is *r*(202) = −0.30, *P* < 0.01, consistent with significant correlation. The data presented are ± SD. We include the 95% confidence band.

**Fig. 7:**
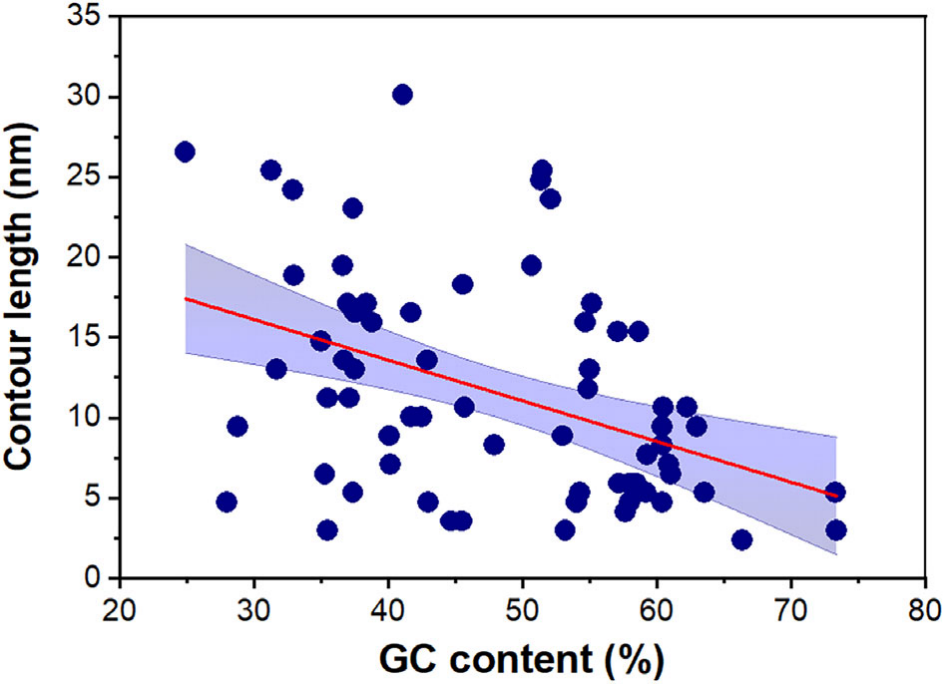
Contour length vs GC content from the predicted mRNA secondary structures in related species. Blue circles correspond to values obtained from homologous genes using Vienna RNA, *N* = 70. The plot includes the linear fit (red line) (*y* = *a + bx*) with *a* = 23.7 ± 3.2 nm and *b* = −0.25 ± 0.06 nm/% GC. The Pearson correlation coefficient is *r*(68) = −0.42, *P* < 0.01, consistent with significant correlation. The data presented are ± SD. We include the 95% confidence band.

Since both the length of the 3′‐UTRs and their whole GC content could impact on the overall structure of RNA molecules, we decided to investigate whether these two parameters have any correlation with the separation between both ends of mRNA molecules. As can be found in Figs S5 and S6, the 3′‐UTR average length is not correlated with their *C*_L_ values. Thus, the 3′‐UTR length no impact on the *C*_L_ value. However, the GC content negatively correlates with a lower *C*_L_ value. Figure 6 shows that higher GC content negatively correlates with a lower *C*_L_ value (Pearson correlation coefficient *r*(202) = −0.30, *P* < 0.01; linear fit to the data *a =* 18.6 ± 2 nm and *b =* −0.18 ± 0.04 nm/% GC) (± SD). Likewise, when only the homologous genes were analyzed, a higher GC content negatively correlates with a lower *C*_L_ value (Fig. 7) (*r*(68) = −0.42, *P* < 0.01; *a =* 23.7 ± 3.2 nm and *b =* −0.25 ± 0.06 nm/% GC) (± SD).

## Discussion

There is no doubt about the necessity of an effective circularization of the ends of mRNA molecules to increase their translation efficiency [7]. However, our working hypothesis is that the intrinsic thermodynamic properties given by the sequence of RNA molecules determines the extend of that circularization (*C*_L_ size), and therefore, their rate of translation initiation efficiency. In our hypothesis, instead of a mRNA circularization induced by RNA binding proteins [5, 6], the inherent proximity of both ends in the mRNA molecules promotes the initiation of cap‐dependent protein synthesis by favoring the recognition of the 5′‐cap and the 3′ poly(A) tail by eIF4E and PABP, respectively [7, 48]. Similarly, in uncapped, non‐polyadenylated positive‐single RNA stranded plant viruses, the initiation of protein synthesis is driven by 3′ cap‐independent translation enhancers (3′ CITEs) through base‐pairing with complementary sequences in the 5′ UTR inducing an effective circularization that favors the recruiting the initiation factor eIF4F and PABP [49]. Moreover, positive‐single‐stranded RNA genomes of Flaviviruses (like Dengue and Zika viruses, ~ 11 kb) require an active self‐induced circularization for full replication efficiency [50]. Therefore, small *C*_L_ values of mRNA molecules would favor their translation efficiency, whereas large separations would decrease it. Strikingly, we found larger *C*_L_ values than previously reported [8, 9] (Fig. 3). Furthermore, full native mRNA sequences show much larger variations than those of random sequences that could not be explained just by statistical variations, and native RNA sequences present a lower MFE values than their correspondent random sequence [51, 52]. Therefore, we reasoned that there must be a biological impact related to the variability in *C*_L_ values we observed. In other words, statistical variations are not big enough to explain the variations observed in native sequences, with remarkably high confidence.

In this regard, the efficiency of protein synthesis is one of the important internal features that allows organisms to adapt and survive to changes in the external conditions. Therefore, the stability of phenotypes could be a feature that might depend on small distances between the ends of mRNA molecules. In line with this, *GB* and *CR*, the two species with the smallest *C*_L_ values (4.8 ± 0.7 nm and 6.4 ± 0.9 nm, respectively), have an increased ability to cope with adverse conditions or environmental changes. For example, *GB*, has an impressive capacity to resist serious pests and diseases, as well as a high tolerance to city smoke and industrial fumes [53, 54], whereas *CR* shows a strong phenotypic stability after exposure to either high CO_2_ concentrations during 1000 generations [55] or to CO_2_ limiting conditions [56]. Moreover, viroids of the Avsunviroidae and Pospiviroidae families, whose genomes are composed of circular ssRNA [57], show a lack of divergence and diversification [58]. Conversely, larger *C*_L_ values could favor variability upon stress or pressure, leading to phenotypical instability, and perhaps, divergency. Although the ability to survive or diverge depends on a combination of several characteristics under the appropriate environmental and intrinsic conditions, we expected to obtain some insight by considering the length of the exterior loop as one of the intrinsic conditions to take into account. In this regard, if there is a biological impact for the extent of *C*_L_ values, an upper limit in the distance between ends of a mRNA molecule should exist. Using statistical analysis, we determined that *C*_L_ values larger than 17.5 ± 2 nm (± SD) are not favored. In addition, we expected that related species in a similar evolutive process would not show significant differences in their *C*_L_ values, whereas groups with few extant species would show the smaller *C*_L_ values. Supporting these ideas, the eudicotyledones (*HB*/*AT*) and the hominids (*PT/HS*) pairs, which are in similar phylogenetic divergence level, present similar *C*_L_ values (Fig. 5); and *GB*, the most ancient living tree [53, 54] and the only extant species of the Order Ginkgoales have the smallest *C*_L_ values we found. Interestingly, *CR* (*C*_L_ of 6.4 ± 0.9 nm) is the evolutionary predecessor of *VC* (*C*_L_ of 11.3 ± 1.2 nm), and when the *C*_L_ values of their homologous genes are compared, no statistical differences could be found (Fig. 5). However, when the *C*_L_ values of their heterologous genes are compared, they are clearly different. Indeed, homologous genes maintain similar *C*_L_ values (Fig. 5). Thus, our results might suggest that heterologous genes could be involved in the ability of species to diverge, as their *C*_L_ values are increased in comparison to that of homologous genes.

Finally, it is important to note that all analyses were performed using full‐length mRNA sequences, all of them contained their respective 5′‐UTR and 3′‐UTR. This means that all sequences used to calculate their *C*_L_ values started with their transcription starting nucleotide and included the typical polyadenylation signal (PAS) for that species, located downstream of the coding sequence. No particular attention was put on alternative PAS, as we only wanted to analyze full‐length mRNAs. Furthermore, it could be argued that the poly‐A tail should increase the size of the *C*_L_ values, although in essence, this is true, the cytoplasmic PABP (PABPc) has the ability to bind to a stretch of 12 As with high affinity [59, 60] while covering 25 nt [61]. Therefore, it is very likely that PABPc could bind to the initial segment of the poly‐A tail while interacting with the 3′ end portion of the exterior loop, generating the appropriate distance to interact with eIF4F, located in the 5′ end portion of the exterior loop, thus making the entire size of the poly‐A tail irrelevant, at least for the initiation of translation.

## Conclusion

A fundamental question in biology is to predict how changes in genotypes could result in changes in phenotypes. Here, we show that the variations in the distance between ends of native mRNA molecules, represented here by the *C*_L_ of the exterior loop, are somewhat larger than previously reported using housekeeping and highly expressed genes. Considering that end‐to‐end separation of mRNA molecules could impact the initiation of transcription, our results suggest that the variability in *C*_L_ could be related to phenotypical stability.

In this regard, it is important to note that phenotypical stability, which could modulate the ability of a species to diverge or survive, does not depend on just one characteristic. Instead, it depends on a combination of several characteristics under the appropriate environmental and intrinsic conditions. Here we propose that the length of the exterior loop is one of the intrinsic conditions to be considered.

## Conflict of interest

The authors declare no conflict of interest.

### Peer review

The peer review history for this article is available at https://www.webofscience.com/api/gateway/wos/peer‐review/10.1002/2211‐5463.13877.

## Author contributions

NG did the search, computer simulations of the mRNA molecules and statistical analysis. JAM, EG and JR‐G design the scientific work. All authors contributed to the writing to the manuscript.

## Supporting information


**Fig. S1.** Exterior loop of the minimum free energy mRNA secondary structure.
**Fig. S2.** Minimum free energy secondary structure for 464 nt mRNA of *BG Prepro‐hypertrehalosemic hormone*.
**Fig. S3.** Minimum free energy secondary structure for 997 nt mRNA of *GB Nuclear‐encoded chloroplast chlorophyll a/b binding protein*.
**Fig. S4.** Minimum free energy secondary structure for 2411 nt mRNA of *VC Channelrhodopsin‐2*.
**Fig. S5.** Average contour length vs 3′‐UTR length.
**Fig. S6.** Average contour length vs 3′‐UTR length from homologous genes in related species.
**Table S1.** mRNA molecules studied from 17 model organisms.

## Data Availability

All data generated or analyzed during this study are included in this published article [and its supplementary information files].
