# Supplementary material for: The separation between mRNA‐ends is more variable than expected
Source: FEBS Open Bio. 2024 Sep 3;14(12):1985–95. doi: 10.1002/2211-5463.13877 (PMC11609591; doi:10.1002/2211-5463.13877)
Supplement: Supplementary file 1 — Fig. S1. Exterior loop of the minimum free energy mRNA secondary structure. Fig. S2. Minimum free energy secondary structure for 464 nt mRNA of BG Prepro‐hypertrehalosemic hormone. Fig. S3. Minimum free energy secondary structure for 997 nt mRNA of GB Nuclear‐encoded chloroplast chlorophyll a/b binding protein. Fig. S4. Minimum free energy secondary structure for 2411 nt mRNA of VC Channelrhodopsin‐2. Fig. S5. Average contour length vs 3′‐UTR length. Fig. S6. Average contour length vs 3′‐UTR length from homologous genes in related species. Table S1. mRNA molecules studied from 17 model organisms. [file FEB4-14-1985-s001.docx]

**Supplementary Material**

**The separation between mRNA-ends is more variable than expected**

N. Gerling, J. Alfredo Mendez, E. Gomez and J. Ruiz-Garcia

**Table S1.** **mRNA molecules studied from 17 model organisms.** A) The mRNA names are placed in order of the temporal range of their first ancestors' appearance. B) Homologous mRNA molecules studied from related organisms. The green algae *C. reinhardtii* with *V.* *carteri*, the fishes *L. camtschaticum* with *D. rerio*, the eudicotyledones *H. brasiliensis* with *A. thaliana* and the hominids *P. troglodytes* with *H. sapiens*.

**A**

| **Organism** | **mRNA name** | **Total length (nt)** | **GenBank** |
| --- | --- | --- | --- |
| *H. salinarum (HbS)* | blp bacterioopsin-linked protein blp^a^  Sod2 superoxide dismutase 2^a^  thrC1 threonine synthase^a^  mcmA1 methylmalonyl-CoA mutase subunit A^a^ | 503  644  1302  1766 | NC_010364.1  NC_010364.1  NC_010364.1  NC_010364.1 |
| *C. reinhardtii (CR)* | Putative copper chaperone Atx1 mRNA  LciA mRNA for low-CO2 inducible protein LCIA  Atp2 (atpB) mRNA  Lcr1 mRNA for low-CO2 inducible Myb transcription factor LCR1  Protein S5 precursor (Prps5) mRNA  Thioredoxin f1 (TRXf1) mRNA  Halo-acid dehalogenase-like hydrolase (HDH1) mRNA  Nitrite transporter NAR1 mRNA  Chloroplast ATP-binding protein (Sabc) mRNA  Chloroplast sulfate permease (SulP2) mRNA  Chloroplast beta carbonic anhydrase (Cah6) mRNA  Possible membrane protein, low CO2-induced mRNA  delta-aminolevulinic acid dehydratase (alad) mRNA  gliding motility related CaM kinase mRNA  Lci6 mRNA for low-CO2 inducible protein LCI6  Thioredoxin x (TRXx) mRNA  thioredoxin o (TRXo) mRNA  cytosolic thioredoxin h2 (TRXh2) mRNA  thioredoxin y (TRXy) mRNA, complete cds  Chloroplast sulfate-binding protein (Sbp) mRNA | 391  1917  2599  3195  2303  1879  1351  2044  2253  1863  2452  1294  1717  2002  1931  1161  1176  1126  1313  1853 | AF280056.1  AB168092.1  X61624.1  AB168090.1  AY093615.1  AY184800.1  AY672644.1  AF149737.1  AY536252.1  AY536251.1  AY463239.1  U31976.1  U19876.1  AY348297.1  AB168091.1  AY184799.1  AY184798.1  AY184797.1  AY184796.1  AY536253.1 |
| *V. carteri (VC)* | Small cysteine-rich extracellular protein VCRP1 precursor mRNA  Channelrhodopsin-2 mRNA  GDP dissociation inhibitor protein GDIV1p (gdiV1) mRNA  Retinoblastoma-related protein 1 mRNA  channelrhodopsin-1 mRNA  small cysteine-rich extracellular protein VCRP2 precursor mRNA  somatic regenerator RegA (regA) | 1243  2411  2519  3656  2694  1027  6725 | DQ521274.1  EU285660.1  U62866.1  EU366288.1  EU285658.1  EU143653.1  AF106963.1 |
| *L. camtschaticum (LC)* | CNP mRNA for C-type natriuretic peptide  LjMA1 mRNA for muscle actin  Chitinase (chit) mRNA  ubiquitin-conjugating enzyme UBE2A mRNA  wingless-type MMTV integration site family member 1 (Wnt1) mRNA  nk2-1 family homeobox c (Nkx2-1/2-4C) mRNA  LjMA2 mRNA for muscle actin  mRNA for aldolase (EJM8)  mRNA for aldolase (EJL3U)  NF-kappaB mRNA  I kappa B-epsilon (IkBe)  CD29-like protein mRNA | 990  1466  2797  1289  1313  2925  2472  2221  1761  4764  3625  3698 | AB205156.1  AB076674.1  EU741679.1  KP203887.2  KT897931.1  KT897927.1  AB052654.2  D38620.1  D38619.1  KY652748.1  KC335304.1  GU013762.1 |
| *P. imperator (PI)* | mRNA for hemocyanin subunit 6 (hc6 gene)  mRNA for hemocyanin subunit 3b  mRNA for hemocyanin subunit 5b (hc5b gene)  mRNA for hemocyanin subunit 3a (hc3a gene)  mRNA for hemocyanin subunit 5a (hc5a gene)  mRNA for hemocyanin subunit 3c (hc3c gene) | 1995  2063  2189  2494  2079  2246 | FN424083.1  FN424082.1  FN424086.1  FN424079.1  FN424084.1  FN424081.1 |
| *B. germanica (BG)* | Prepro-hypertrehalosemic hormone mRNA  Hypertrehalosemic hormone receptor mRNA  1,4-alpha-D-glucan glucanohydrolase precursor (bgtg-1) mRNA  ace2 type acetylcholinesterase mRNA  triosephosphate isomerase (tpi) mRNA  mRNA for Yorkie-L (yki gene)  mRNA for Hippo (hpo gene)  mRNA for FoxO protein  mRNA for Na/K-ATPase subunit beta 1 (nrv1 gene)  receptor for activated protein kinase C-like (RACK1) mRNA  mRNA for ecdysone inducible protein 75 isoform B (e75 gene)  mRNA for ecdysone inducible protein 75 isoform A (e75 gene)  mRNA for fruitless (fru gene)  mRNA for squid, variant G (sqd gene)  ace1 type acetylcholinesterase mRNA  mRNA for glutathione S-transferase (gstd1 gene)  mRNA for Yorkie-S (yki gene) | 464  1745  2035  2430  1233  1700  2079  2266  1145  1147  3102  3232  1158  1445  2683  956  1622 | FJ943774.1  GU591493.1  AY945930.1  DQ288847.1  DQ885469.1  HF969252.1  HF969251.1  HE648216.1 HE795995.1  DQ885470.1  AM238654.1 AM238653.1 FN429764.1 FM875794.1  DQ288249.1  AM778448.1  HF969253.1 |
| *P. sylvestris (PS)* | glyceraldehyde-phosphate dehydrogenase mRNA  CCAAT-box binding factor HAP3-like protein (HAP3A) mRNA  mRNA for ornithine aminotransferase (dOAT gene)  NAD+-dependent glyceraldehyde-3-phosphate dehydrogenase  glyceraldehyde-3-phosphate dehydrogenase (GapC1) mRNA | 1454  812  1967  1615  1304 | L26923.1  JF280795.1  AM228955.1 L32560.1 L07501.1 |
| *G. biloba (GB)* | Defensin precursor mRNA  Nuclear-encoded chloroplast chlorophyll a/b binding protein mRNA  WD40-repeat protein (WD40) mRNA  3-hydroxy-3-methylglutaryl coenzyme A reductase mRNA  glyceraldehyde-phosphate dehydrogenase mRNA  ginkbilobin-2 precursor mRNA  mRNA for putative auxin response factor 6/8 (arf6/8 gene)  lipid transfer protein precursor, mRNA | 506  997  1421  2206  1192  712  3450  700 | AY695796.1  L23107.1  KJ630503.1  AY741133.1  L26924.1  DQ496113.1 FN433179.1  DQ836633.1 |
| *G. gallus (GG)* | Preproghrelin mRNA  Heme oxygenase 1 (hmox1) mRNA  Vesicular glutamate transporter 2 (VGLUT2) mRNA  EGF/TGF-alpha receptor (c-erbB) mRNA  paraoxonase-2 (PON2) mRNA  PGK mRNA  kinase related protein mRNA  (17.5) mRNA  p94 mRNA for n-calpain-1 large subunit  YB-1 protein mRNA  alpha-3 type IX collagen mRNA  liver ribonuclease A precursor, mRNA  WDR1 protein mRNA  chS-Rex-b mRNA  chS-Rex-s mRNA  protein arginine methyltransferase 4 (PRMT4) mRNA  neural retina growth hormone mRNA  TRF2-interacting telomeric RAP1 protein (RAP1) mRNA | 843  1565  1760  2243  1262  1453  2535  2046  3454  1507  2416  577  3280  3187  1572  1788  690  2399 | AY299454.1  HM237181.1  JF320001.1  M77637.1  L47573.1  L37101.1  M88283.1  M88072.1  D38028.1  L13032.1  M83179.1  DQ395277.1  AF020054.1  U17606.1  U17605.1  KY655811.1  AY373631.1  AY083608.1 |
| *M. domestica (MD)* | Early lactation protein precursor (ELP) mRNA  Sperm protein Sp17 mRNA  Endogenous retrovirus ERV syncytin-Opo1 mRNA  Endogenous retrovirus Opo-Env3-ERV Env3 mRNA  mRNA for Interleukin-6 receptor alpha (IL6R gene)  domestica mRNA for Interleukin-6 (IL6 gene)  anterior pituitary glycoprotein hormone common alpha subunit mRNA  thyroid stimulating hormone beta subunit precursor mRNA  follicle-stimulating hormone beta precursor mRNA  somatotropin precursor mRNA  p21-ras mRNA  beta (2) microglobulin mRNA | 477  1087  2145  2481  2131  1255  755  517  450  813  796  1105 | JN191340.1  AF054290.1  KM235357.1  KM235359.1  LT596680.1  LT596676.1  AY048590.1  AY048589.1  AF406610.1  AF312023.1  Z12125.1  AY125947.1 |
| *Z. mays (ZM)* | Nuclear-encoded mitochondrial F1F0 ATP synthase epsilon subunit  mudrB mRNA  Beta-8 tubulin (tub8) mRNA  Sucrose transporter 2 (SUT2) mRNA  O-methyltransferase mRNA  beta-7 tubulin (tub7) mRNA  beta-6 tubulin (tub6) gene and mRNA  cytochrome P-450 (cyp78) mRNA  putative bifunctional nuclease (nuc gene)  mRNA for putative inositol-3-phosphate synthase (mips2 gene)  mRNA for transcription factor MYB42 (myb42 gene)  mRNA for transcription factor MYB39 (myb39 gene)  mRNA for transcription factor MYB31 (myb31 gene)  mRNA for transcription factor MYB8 (myb8 gene)  mRNA for transcription factor MYB2 (myb2 gene)  harpin binding protein 1 (HrBP1) mRNA | 488  1030  1625  2149  1268  1583  1730  2087  1095  1848  1129  1078  1264  1082  1339  1218 | L39120.1  U14598.1  L10636.1  AY581895.1  L14063.1  L10634.1  L10633.1  L23209.1  AM710418.1  AM295187.1  AM156908.1  AM156907.1  AM156906.1  AM156905.1  AM156904.1  AY388616.1 |
| *H. brasiliensis (HB)* | Copper transport protein ATOX1 (CCH) mRNA  JAZ11 mRNA  MYB transcription factor (MYB) mRNA  JAZ9 mRNA  subtilisin-like serine protease C (SPC) mRNA  subtilisin-like serine protease A (SPA) mRNA  JAZ10 mRNA  JAZ8 mRNA  JAZ7 mRNA  mRNA for latex allergen | 543  914  970  1391  2389  2444  804  526  644  1419 | GU550955.1  KJ001648.1  DQ323739.1  KJ001646.1  KU845304.1  KU845302.1  KJ001647.2  KJ001645.1  KJ001644.1  AJ223038.1 |
| *A. thaliana (AT)* | Thionin (Thi2.2) mRNA  rac GTP binding protein mRNA  Heat shock mRNA  cystathionine beta-lyase mRNA  molybdenum cofactor biosynthesis enzyme (cnx1) mRNA  serine acetyltransferase (SAT1) mRNA  GTP-binding protein mRNA  lipoxygenase mRNA  RNA polymerase subunit (isoform B) mRNA  RNA polymerase subunit (isoform A) mRNA  thionin (Thi2.1) mRNA  recombination and DNA-damage resistance protein (DRT112) mRNA  thaliana rac GTP binding protein Arac10 (Arac10) mRNA  PAC3 mRNA | 718  985  3105  1644  2237  1079  2234  2790  1406  1282  611  705  794  1200 | L41245.1  AF079485.1  U13949.1  L40511.1  L47323.1  L42212.1  L38614.1  L04637.1  L34773.1  L34772.1  L41244.1  M98456.1  AF079485.1  L35241.1 |
| *A. cerana (AC)* | Glutaredoxin 1 (Grx1) mRNA  1-cys thioredoxin peroxidase (Tpx4) mRNA  Arginine kinase (AK) mRNA  Phenoloxidase subunit A3 (PPO) mRNA  superoxide dismutase 2 (SOD2) mRNA  CTL5 (CTL5) mRNA  cuticular protein CPF1 (CPF1) mRNA  cuticle protein 2 (CPR2) mRNA  caspase 1 mRNA  mitogen-activated protein kinase kinase 4 (MKK4) mRNA  triosephosphate isomerase (Tpi) mRNA  ERR (ERR) mRNA  decapentaplegic (Dpp) mRNA  glutathione S-transferase (GSTO1) mRNA  thioredoxin 1 (Trx1) mRNA  CAT (CAT) mRNA  glutathione S-transferase omega 2 (GSTO2) mRNA  thioredoxin 2 (Trx2) mRNA  cytochrome P450 4G11 (CYP4G11) mRNA  delta-class glutathione S-transferase (GSTD) mRNA | 436  931  1650  2293  1003  1197  1021  1134  1328  1634  1894  1671  1652  1040  649  1905  1365  407  2041  1009 | JX844656.2  KJ551847.1  KF772855.1  JX844653.1  JN637476.1  KT808468.1  KJ634544.1  KJ502287.1  KF955542.1  KF017207.1  KP994676.1  KP398511.1  KT750952.1  KF496073.1  JX844651.2  KF765424.1  JX434029.1  JX844649.1  KC243984.1  JF798573.1 |
| *D. rerio (DR)* | flii mRNA for flightless I  fibrosin protein (fbrs)  heat shock cognate (hsc70)  contactin-associated protein-like 2b (cntnap2b)  contactin-associated protein-like 2a beta isoform (cntnap2a)  contactin-associated protein-like 2a alpha isoform (cntnap2a) mRNA  growth hormone receptor a mRNA  myogenin mRNA  QM protein (QM) mRNA  neuropeptide FF-related PQRF precursor (PQRF)  NADPH-cytochrome P450 oxidoreductase  CL2 mRNA  connexin 41.8 (cx41.8) mRNA | 3878  5142  2323  4751  888  4558  2332  1364  769  827  2941  2926  2801 | AB355792.1  KY492383.1  L77146.2  HQ880438.1  HQ880437.1  HQ880436.1  EU649774.1  AF202639.1  AY763500.1  AY092774.1  AY949986.1  EU269066.1  DQ177156.1 |
| *P. troglodytes (PT)* | Hepcidin (HAMP) mRNA  Glycolipid transfer protein (GLTP) mRNA  Gene for non-A non-B hepatitis-associated microtubular mRNA  Dusty protein kinase mRNA  mRNA for Killer-cell Ig-like receptor KIR2DL8 (KIR2DL8 gene)  mRNA for beta1,4-galactosyltransferase 7 (b4Gal-T7 gene)  beta-defensin 104 (DEFB104) mRNA | 391  722  1641  3601  1064  1074  285 | EU076436.1  EF688398.1  D90034.1  AY641092.1  AM279149.2  AM231264.1  EU126867.1 |
| *H. sapiens (HS)* | mRNA for Ubiquitin protein ligase  prostasin mRNA  K+ channel beta-subunit (Kvb1.3) mRNA  neuroendocrine-specific protein C (NSP) mRNA  STAT4 mRNA  ERK3 protein kinase mRNA  FRG1 mRNA  cyclin G2 mRNA  interleukin 8 receptor alpha (IL8RA) mRNA  phosphatase 2A B56-epsilon (PP2A) mRNA  cyclin G1 mRNA  5-HT6 serotonin receptor mRNA  pyruvate dehydrogenase kinase isoenzyme 3 (PDK3) mRNA  pyruvate dehydrogenase kinase isoenzyme 2 (PDK2) mRNA  casein kinase I epsilon mRNA | 2850  1834  3103  1416  2588  3324  1042  1410  2007  3270  1602  1984  1599  1422  1331 | AB056663.2  L41351.1  L47665.1  L10335.1  L78440.1  L77964.1  L76159.1  L49506.1  L19591.1  L76703.1  L49504.1  L41147.1  L42452.1  L42451.1  L37043.1 |

**B**

| **Homolog mRNA name** | **Organisms** | **Total length (nt)** | **CDS protein identity (%)** | **GenBank** |
| --- | --- | --- | --- | --- |
| ubiquitin conjugating enzyme E2 | *C. reinhardtii (CR)*  *V.* *carteri (VC)* | 2282  1638 | 25 | [XP_001690015.1](https://www.ncbi.nlm.nih.gov/protein/159463570)  [XP_002953636.1](https://www.ncbi.nlm.nih.gov/protein/302844191) |
| thioredoxin-like protein (TRX10) | *C. reinhardtii (CR)*  *V.* *carteri (VC)* | 1009  1456 | 27 | [XP_001690017.1](https://www.ncbi.nlm.nih.gov/protein/159463574)  [XP_002958831.1](https://www.ncbi.nlm.nih.gov/protein/302854651) |
| glyceraldehyde-3-phosphate dehydrogenase (GAP3) | *C. reinhardtii (CR)*  *V.* *carteri (VC)* | 1930  1627 | 45 | [XP_001689871.1](https://www.ncbi.nlm.nih.gov/protein/159463282)  [XP_002956882.1](https://www.ncbi.nlm.nih.gov/protein/302850712) |
| p53-induced protein 8 | *C. reinhardtii (CR)*  *V.* *carteri (VC)* | 2684  1507 | 69 | [XP_001690067.1](https://www.ncbi.nlm.nih.gov/protein/159463674)  [XP_002955375.1](https://www.ncbi.nlm.nih.gov/protein/302847683) |
| vacuolar ATP synthase subunit H (ATPvH) | *C. reinhardtii (CR)*  *V.* *carteri (VC)* | 2603  1808 | 82 | [XP_001689562.1](https://www.ncbi.nlm.nih.gov/protein/159462664)  [XP_002955475.1](https://www.ncbi.nlm.nih.gov/protein/302847883) |
| peroxiredoxin, type II (PRX5) | *C. reinhardtii (CR)*  *V.* *carteri (VC)* | 1180  1336 | 83 | [XP_001689455.1](https://www.ncbi.nlm.nih.gov/protein/159462450)  [XP_002956367.1](https://www.ncbi.nlm.nih.gov/protein/302849676) |
| 20S proteasome alpha subunit D (POA4) | *C. reinhardtii (CR)*  *V.* *carteri (VC)* | 1267  1025 | 91 | [XP_001689587.1](https://www.ncbi.nlm.nih.gov/protein/159462714)  [XP_002955451.1](https://www.ncbi.nlm.nih.gov/protein/302847835) |
| translation initiation factor 4E (eif4E) | *C. reinhardtii (CR)*  *V.* *carteri (VC)* | 2032  1696 | 91 | [XP_001693235.1](https://www.ncbi.nlm.nih.gov/protein/159470175)  XP_002958375.1 |
| 26S proteasome regulatory subunit (RPN11) | *C. reinhardtii (CR)*  *V.* *carteri (VC)* | 1964  1419 | 93 | [XP_001689423.1](https://www.ncbi.nlm.nih.gov/protein/159462386)  [XP_002955275.1](https://www.ncbi.nlm.nih.gov/protein/302847482) |
| ribosomal protein L18a (RPL18a) | *C. reinhardtii (CR)*  *V.* *carteri (VC)* | 1086  1121 | 94 | [XP_001689743.1](https://www.ncbi.nlm.nih.gov/protein/159463026)  [XP_002948508.1](https://www.ncbi.nlm.nih.gov/protein/302833890) |
| histone H2B | *C. reinhardtii (CR)*  *V.* *carteri (VC)* | 571  717 | 96.77 | [XP_001691693.1](https://www.ncbi.nlm.nih.gov/protein/159467014)  XP_002955481.1 |
| histone H4 (HFO24) | *C. reinhardtii (CR)*  *V.* *carteri (VC)* | 1457  653 | 99.03 | [XP_001690685.1](https://www.ncbi.nlm.nih.gov/protein/159464912)  XP_002955420.1 |
| ATP synthase F0 subunit 8 | *L. camtschaticum (LC)*  *D. rerio* *(DR)* | 794  630 | 40.74 | [YP_007517126.1](https://www.ncbi.nlm.nih.gov/protein/459627056)  NP_059335.1 |
| NADH dehydrogenase subunit 6 | *L. camtschaticum (LC)*  *D. rerio* *(DR)* | 580  1868 | 41.14 | [YP_007517133.1](https://www.ncbi.nlm.nih.gov/protein/459627063)  [NP_059342.1](https://www.ncbi.nlm.nih.gov/protein/8395623) |
| NADH dehydrogenase subunit 5 | *L. camtschaticum (LC)*  *D. rerio* *(DR)* | 1971  2112 | 54.48 | YP_007517132.1  NP_059341.1 |
| NADH dehydrogenase subunit 1 | *L. camtschaticum (LC)*  *D. rerio* *(DR)* | 1204  1396 | 65.82 | [YP_007517122.1](https://www.ncbi.nlm.nih.gov/protein/459627052)  [NP_059331.1](https://www.ncbi.nlm.nih.gov/protein/8395612) |
| cytochrome c oxidase subunit III | *L. camtschaticum (LC)*  *D. rerio* *(DR)* | 1627  1694 | 80.08 | [YP_007517128.1](https://www.ncbi.nlm.nih.gov/protein/459627058)  [NP_059337.1](https://www.ncbi.nlm.nih.gov/protein/8395618) |
| cytoplasmic actin | *L. camtschaticum (LC)*  *D. rerio* *(DR)* | 1921  1664 | 98.4 | [BAB41207.1](https://www.ncbi.nlm.nih.gov/protein/13699190)  [NP_571106.2](https://www.ncbi.nlm.nih.gov/protein/1488192366) |
| E3 SUMO-protein ligase SIZ1-like | *H. brasiliensis (HB)*  *A. thaliana (AT)* | 2903  3331 | 65.91 | XP_021644935.1  [AAU00414.1](https://www.ncbi.nlm.nih.gov/protein/51339279) |
| Two pore calcium channel protein 1 | *H. brasiliensis (HB)*  *A. thaliana (AT)* | 3075  2500 | 70.65 | XP_021685070.1  [BAB55460.1](https://www.ncbi.nlm.nih.gov/protein/14041819) |
| ribosomal protein L20 | *H. brasiliensis (HB)*  *A. thaliana (AT)* | 731  1664 | 80.34 | [YP_004327685.1](https://www.ncbi.nlm.nih.gov/protein/326909416)  [NP_051082.1](https://www.ncbi.nlm.nih.gov/protein/7525056) |
| ATP-dependent Clp protease proteolytic subunit | *H. brasiliensis (HB)*  *A. thaliana (AT)* | 642  1516 | 83.59 | YP_004327687.1  NP_051083.1 |
| ribosomal protein S4 (chloroplast) | *H. brasiliensis (HB)*  *A. thaliana (AT)* | 906  798 | 88.56 | [YP_004327664.1](https://www.ncbi.nlm.nih.gov/protein/326909395)  NP_051061.1 |
| NADH dehydrogenase subunit 3 | *H. brasiliensis (HB)*  *A. thaliana (AT)* | 1272  428 | 90.83 | [YP_004327667.1](https://www.ncbi.nlm.nih.gov/protein/326909398)  [NP_051064.1](https://www.ncbi.nlm.nih.gov/protein/7525038) |
| ATP synthase CF1 epsilon subunit | *H. brasiliensis (HB)*  *A. thaliana (AT)* | 725  610 | 91.67 | [YP_004327668.1](https://www.ncbi.nlm.nih.gov/protein/326909399)  [NP_051065.1](https://www.ncbi.nlm.nih.gov/protein/7525039) |
| photosystem I subunit IX | *H. brasiliensis (HB)*  *A. thaliana (AT)* | 768  454 | 95.45 | [YP_004327682.1](https://www.ncbi.nlm.nih.gov/protein/326909413)  NP_051079.1 |
| photosystem II protein M | *H. brasiliensis (HB)*  *A. thaliana (AT)* | 813  181 | 97.06 | [YP_004327647.1](https://www.ncbi.nlm.nih.gov/protein/326909378)  [NP_051053.1](https://www.ncbi.nlm.nih.gov/protein/7525027) |
| V-type proton ATPase 16 kDa proteolipid subunit | *H. brasiliensis (HB)*  *A. thaliana (AT)* | 736  665 | 98.18 | [XP_021659332.1](https://www.ncbi.nlm.nih.gov/protein/1217038718)  [AAA99937.1](https://www.ncbi.nlm.nih.gov/protein/926937) |
| photosystem II protein D2 | *H. brasiliensis (HB)*  *A. thaliana (AT)* | 1583  1613 | 98.58 | [YP_004327657.1](https://www.ncbi.nlm.nih.gov/protein/326909388)  [NP_051054.1](https://www.ncbi.nlm.nih.gov/protein/7525028) |
| dopamine receptor D4 | *P. troglodytes (PT)*  *H. sapiens (HS)* | 1639  1589 | 92.64 | [XP_016775504.1](https://www.ncbi.nlm.nih.gov/protein/1034085954)  [NP_000788.2](https://www.ncbi.nlm.nih.gov/protein/32483397) |
| hemoglobin subunit delta | *P. troglodytes (PT)*  *H. sapiens (HS)* | 927  927 | 99.32 | [XP_001162045.2](https://www.ncbi.nlm.nih.gov/protein/332835679)  [NP_000510.1](https://www.ncbi.nlm.nih.gov/protein/4504351) |
| Arginine vasopressin receptor 1A | *P. troglodytes (PT)*  *H. sapiens (HS* | 2502  2493 | 99.52 | [XP_016778615.1](https://www.ncbi.nlm.nih.gov/protein/1034095037)  [NP_000697.1](https://www.ncbi.nlm.nih.gov/protein/4502331) |
| cytidine deaminase (CDA) | *P. troglodytes (PT)*  *H. sapiens (HS)* | 3056  892 | 100 | [XP_001161389.1](https://www.ncbi.nlm.nih.gov/protein/114554458)  [AAA57254.1](https://www.ncbi.nlm.nih.gov/protein/598149) |
| glutamate-cysteine ligase | *P. troglodytes (PT)*  *H. sapiens (HS)* | 1620  1610 | 100 | [XP_513572.3](https://www.ncbi.nlm.nih.gov/protein/332809547)  [AAA65028.1](https://www.ncbi.nlm.nih.gov/protein/530137) |
| hemoglobin subunit beta | *P. troglodytes (PT)*  *H. sapiens (HS)* | 627  626 | 100 | [XP_508242.1](https://www.ncbi.nlm.nih.gov/protein/55635219)  [NP_000509.1](https://www.ncbi.nlm.nih.gov/protein/4504349) |

**
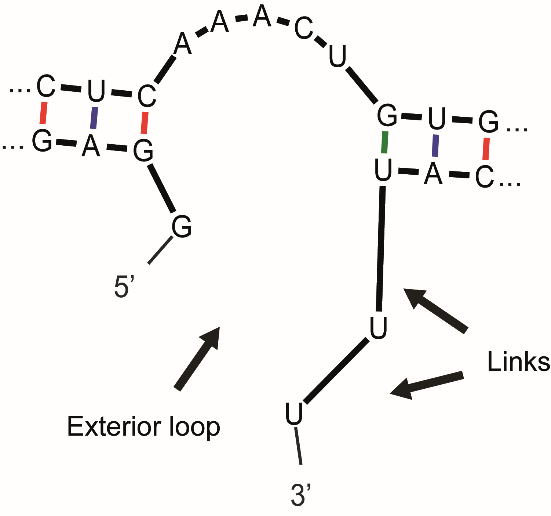
**

**Figure S1.** **Exterior loop of the minimum free energy mRNA secondary structure.** The structure corresponds to mRNA of *HS ubiquitin protein ligase* predicted by mfold. The contour length (*C_L_*) is given by the total number of links in the exterior loop (*L*= 11) multiplied by the distance between nucleotides (*d*= 0.59 nm) giving 6.49 nm in this case.

**
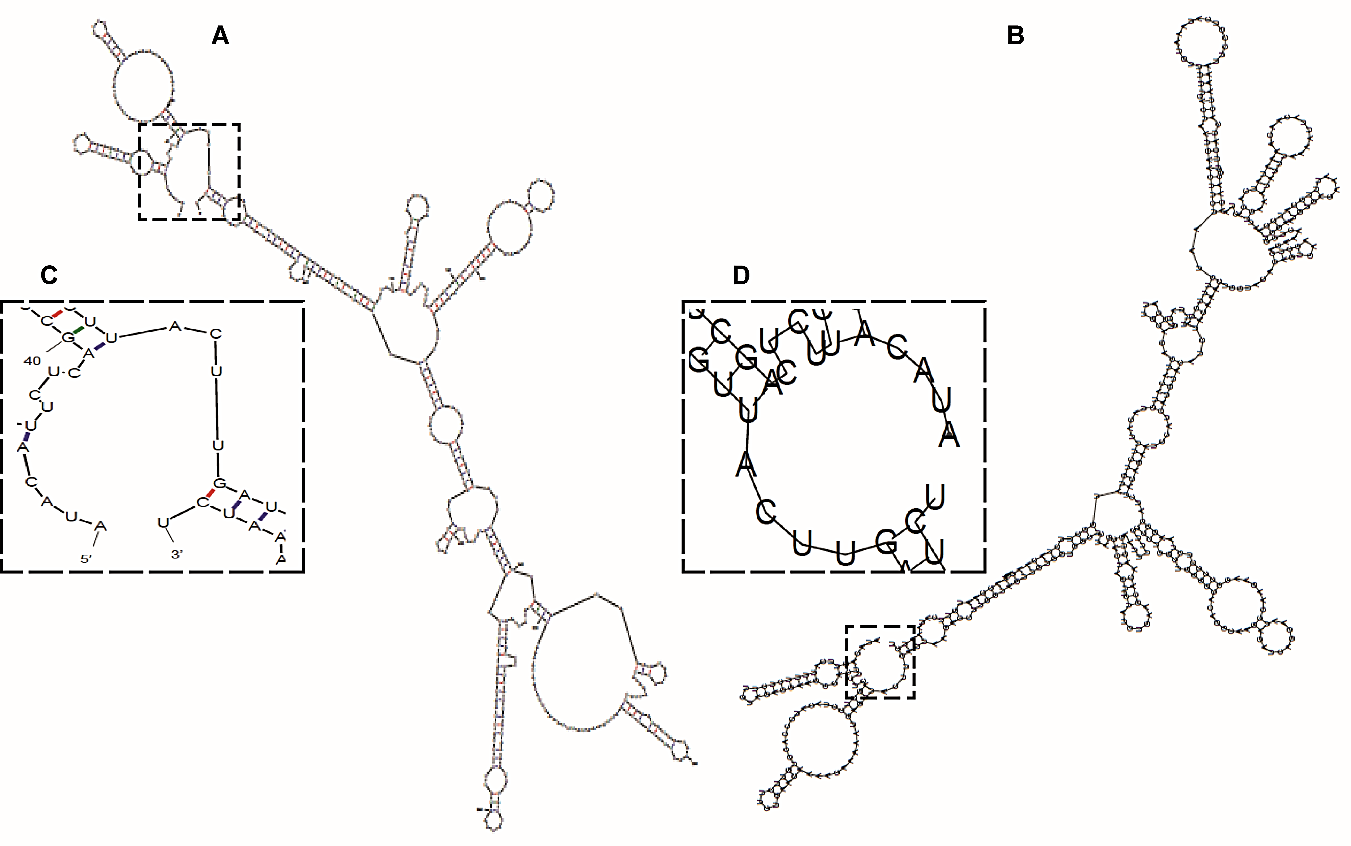
**

**Figure S2. Minimum free energy secondary structure for 464 nt mRNA of *BG Prepro-***

***hypertrehalosemic hormone.*** Obtained by (A) mfold and (B) Vienna RNA algorithms. The

exterior loop is quite similar despite differences on their secondary structure. (C) and (D) zooms

of the exterior loop of (A) and (B), respectively.

**
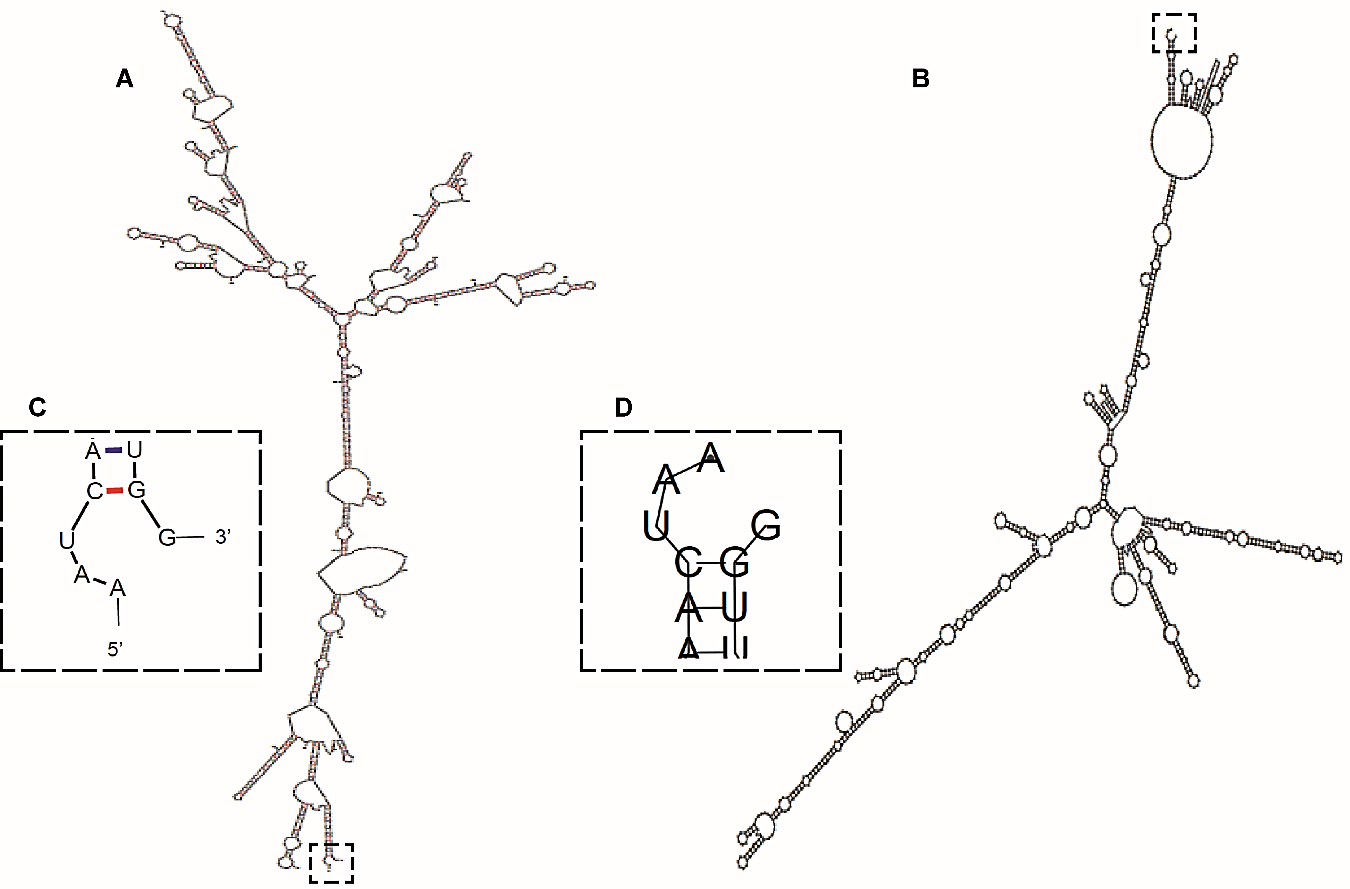
**

**Figure S3. Minimum free energy secondary structure for 997 nt mRNA of *GB Nuclear-encoded chloroplast chlorophyll a/b binding protein.*** Obtained by (A) mfold and (B) Vienna RNA algorithms. The exterior loop is quite similar despite differences on their secondary structure. (C) and (D) zooms of the exterior loop of (A) and (B), respectively.

**
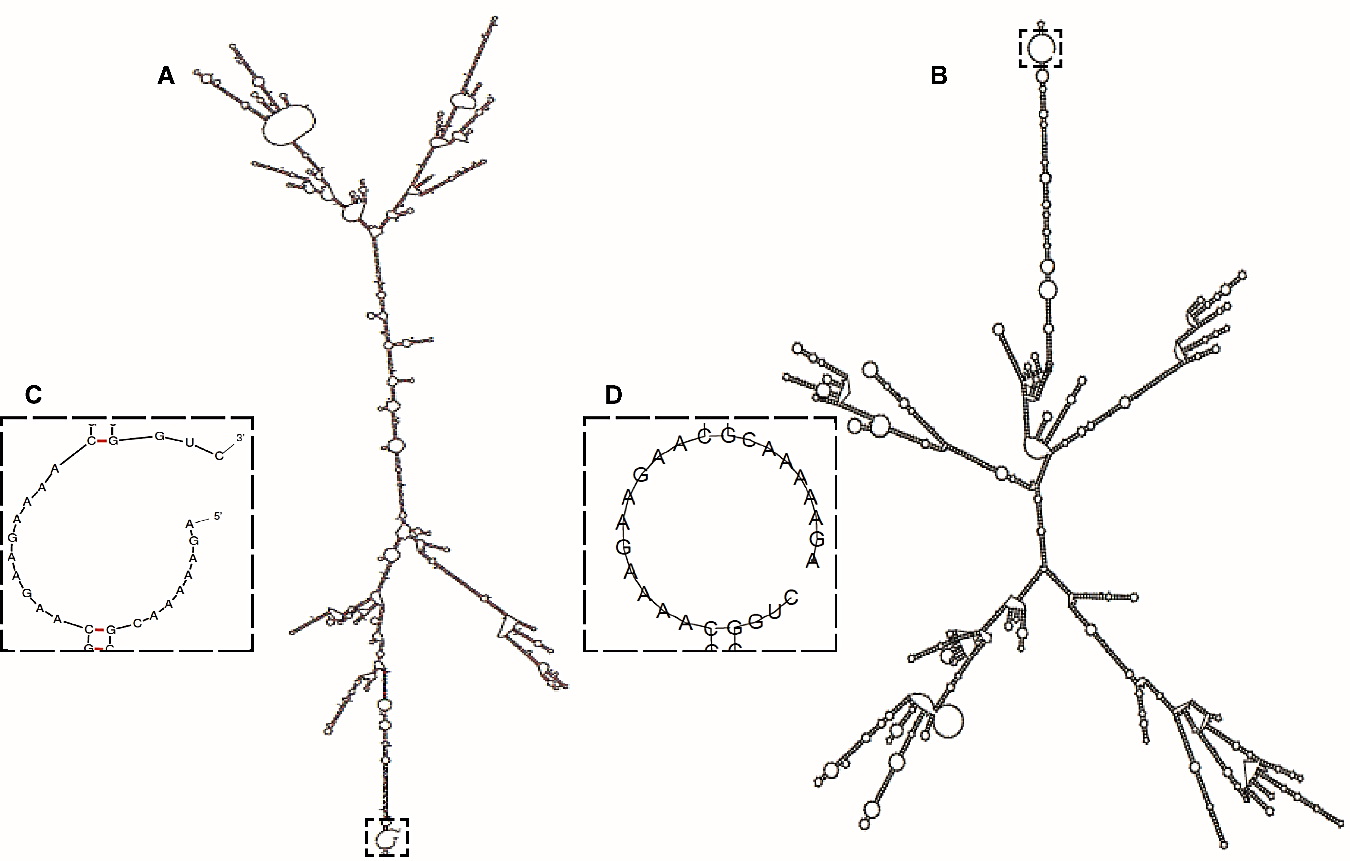
**

**Figure S4. Minimum free energy secondary structure for 2411 nt mRNA of *VC Channelrhodopsin-2.*** Obtained by (A) mfold and (B) Vienna RNA algorithms. The exterior loop is quite similar despite differences on their secondary structure. (C) and (D) zooms of the exterior loop of (A) and (B), respectively.

**
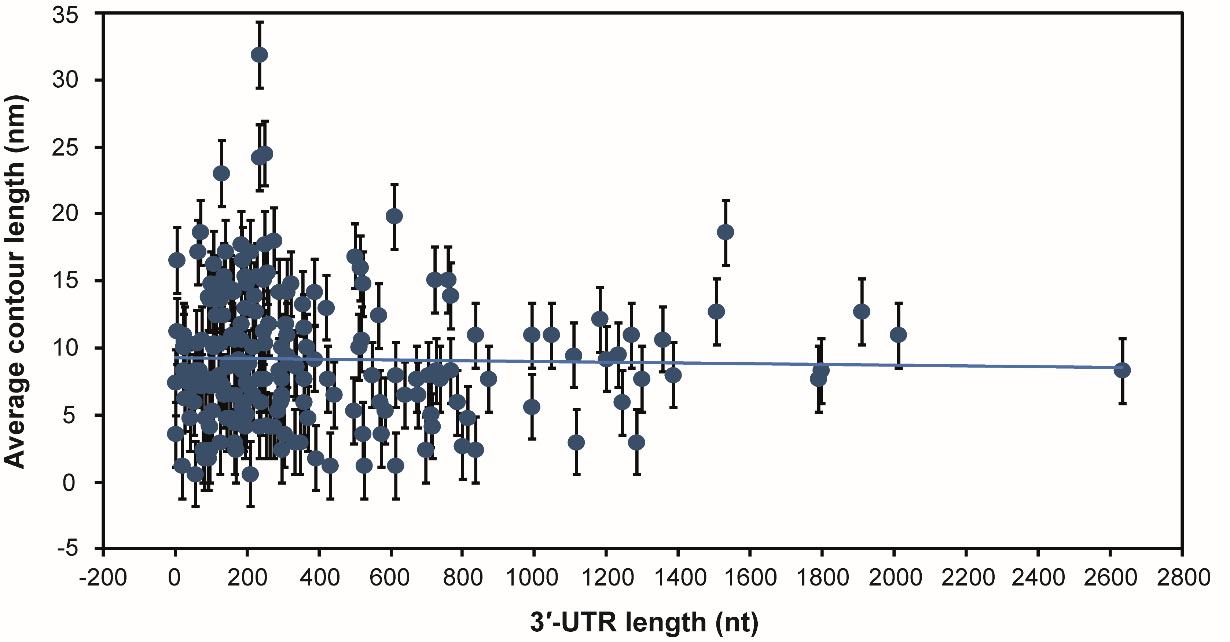
**

**Figure S5.** **Average contour length vs 3**′-**UTR length.** The error bars represent the typical variations obtained with mfold and Vienna RNA. The solid line is a linear fit (*y= a + bx*) with *a=* 9.26 ± 0.47 nm and *b=* - 0.0002 ± 0.0008 nm/nt. The Pearson correlation coefficient is *r*(202)= -0.02, *p=* 0.74, consistent with no correlation.

**
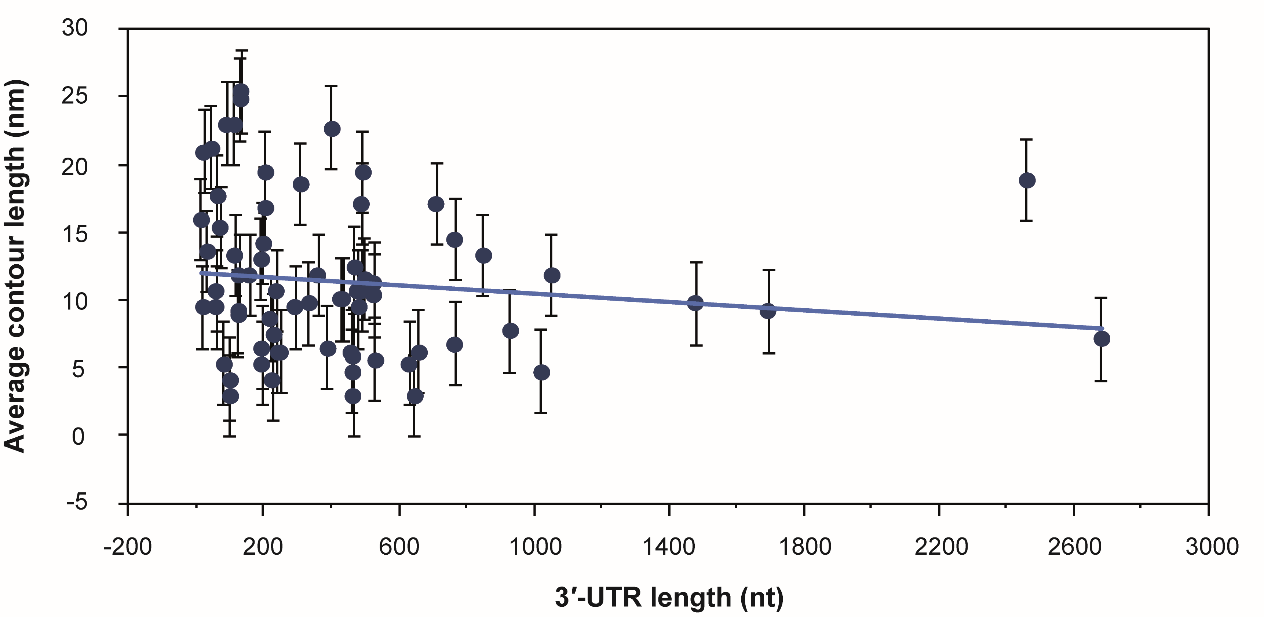
**

**Figure S6. Average contour length vs 3′**-**UTR length from homologous genes in related species.** The error bars represent the typical variations obtained with mfold and Vienna RNA. The solid line is a linear fit (*y= a + bx*) with *a=* 11.9 ± 0.9 nm and *b=* - 0.001 ± 0.001 nm/nt. The Pearson correlation coefficient is *r*(68)= -0.12, *p=* 0.28, consistent with no correlation.
